# Author’s reply

**Published:** 2011

**Authors:** Rohit Shetty

**Affiliations:** Narayana Nethralaya, 121/C, Rajajinagar, Bangalore, Karnataka, India

Dear Editor,

We thank Parthasarthy[1] for his interest in and comments on our paper on Intacs in Indian eyes with keratoconus.[2] Eyes with pachmetry > 400 μm had greater improvement in the sphere and mean refractive spherical equivalent (MRSE) values than thinner corneas.[3] In our series, corneas with <400 μm (*n* = 7) achieved good flattening by the end of 3 months and these eyes showed regression at 6 months and attained the reported spherical equivalent at 1 month. This could be because of biomechanically weaker corneas which were unable to sustain the same effect after 3 months; we noticed better results with “stronger/thicker cornea.” We, however, do not have data (biomechanics) to support this hypothesis.

We had investigated for vernal kerato conjunctivitis prior to the implantation of Intacs; patients with vernal keratoconjunctivitis were treated. All patients were counseled for eye rubbing prior to Intacs implantation. None of these patients had collagen cross-linking prior to or after Intacs implantation.
